# The relationship between biomechanics of pharyngoesophageal segment and tracheoesophageal phonation

**DOI:** 10.1038/s41598-019-46223-7

**Published:** 2019-07-05

**Authors:** Teng Zhang, Ian Cook, Michał Szczęśniak, Julia Maclean, Peter Wu, Duong Duy Nguyen, Catherine Madill

**Affiliations:** 1 Biomechanics Laboratory, Li Ka Shing Faculty of Medicine, Hong Kong, Hong Kong; 2 Faculty of Medicine, The University of New South Wales; Department of Gastroenterology, Sydney, Australia; 3 Cancer Care Centre, St George Hospital, Sydney, Australia; 4 Voice Research Laboratory, The University of Sydney, Sydney, Australia

**Keywords:** Radiography, Outcomes research

## Abstract

This study examined the relationship between biomechanical features of the pharyngoesophageal (PE) segment, acoustic characteristics of tracheoesophageal (TE) phonation, and patients’ satisfaction with TE phonation. Fifteen patients using TE phonation after total laryngectomy completed the Voice Symptom Scale (VoiSS) and underwent acoustic voice analysis for cepstral peak prominence (CPP) and relative intensity. High resolution manometry (HRM) combined with videofluoroscopy was used to evaluate PE segment pressure and calculate the pressure gradient (ΔP), which was the pressure difference between the upper oesophagus and a point two centimetres above the vibrating PE segment. The upper oesophageal sphincter (UOS) minimal diameters were measured by Endolumenal Functional Lumen Imaging Probe (EndoFLIP). HRM detected rapid pressure changes at the level of the 4th – 6th cervical vertebra. CPP, relative intensity, and ΔP were significant predictors of satisfactory TE phonation. ΔP was a significant predictor of CPP and intensity. Minimal UOS diameter was a significant predictor of relative intensity of TE phonation. In two patients with unsuccessful TE phonation, endoscopic dilatation subsequently restored TE phonation. These findings suggest that sufficient ΔP and large UOS diameter are required for satisfactory TE phonation. Endoscopic dilatation increasing UOS diameter may provide a new approach to treat unsuccessful TE phonation.

## Introduction

Verbal communication is one of the most important forms of human interaction^1^. In normal laryngeal phonation, the vocal folds produce a voice signal with well-defined harmonic structures^2^ which are the key component of normal voice quality^3^. In patients with advanced laryngeal cancer who are treated by total laryngectomy, the mechanisms of sound production change permanently. Studies have shown that patients experience a reduced quality of life as a result of their communication impairment after total laryngectomy^4^.

The tracheoesophageal (TE) voice is currently the gold standard for voice restoration in laryngectomy patients^5^. This involves surgical placement of a silicone valve called the TE voice prosthesis between the trachea and the oesophagus. When the tracheostoma is intentionally occluded for phonation, the voice prosthesis allows one-way pulmonary air passage from the trachea through to the oesophagus^6^. The airflow then sets the cervical oesophagus and lower hypopharynx into vibration, generating an acoustic signal^7,8^. This vibrating part, referred to as the pharyngoesophageal (PE) segment, is located in the lower third of the neck, corresponding to cervical vertebrae from C5 to C7^9^. Given that voice characteristics depend on biomechanics of the vibration source^10^, the voice output of TE phonation is influenced by structure and function of the PE segment as a result of the surgical techniques and the patient’s specific anatomy^11^. Research has attempted to investigate the relationship between various characteristics of the PE segment and the resulting TE phonation^12^. Currently, there is limited understanding of how variations in PE dimension, vibratory characteristics, air pressure, and airflow affect TE phonation. Whilst perceptual voice quality, patient satisfaction and quality of life outcomes related to TE phonation have been documented^13^, establishing objective outcome measures such as appropriate biomechanical or acoustic analysis is essential for multidimensional evaluation of the efficacy of any interventions to improve the quality of TE phonation.

Unlike laryngeal phonation where the vocal folds can be observed using laryngoscopy, the whole PE segment cannot be visualized directly except for the visible neoglottis that can be seen via high-speech endoscopy^14^ and videostrobosopy^15^. Therefore, the PE segment has been assessed by a number of parameters such as vibratory characteristics^6^, dimension^16^, position of the PE segment prominence related to the anterior^17^ and posterior pharyngeal walls^16^, intraluminal pressure^18^, and PE geometry^19^. A range of methods have been used to investigate the characteristics of the vibrating PE segment during TE phonation including videofluoroscopy^17^, manometry^20^, and acoustic analysis^21^.

### Videofluoroscopy

This method has proven useful in identifying the location^9^ and measuring the dimensions^16^ of the PE segment. It has also been used to correlate the anatomical features of the PE segment and the resulting perceptual voice quality^22^. van As *et al*.^17^ found significant correlation between TE voice quality and the minimal distance between the PE segment prominence and anterior pharyngeal wall at rest and during phonation. In contrast, Takeshita *et al*.^16^ suggested that TE voice quality was correlated with the anteroposterior distance between the PE segment prominence and the posterior pharyngeal wall in both resting and phonation. As such, there is conflicting evidence regarding the exact association between the quality of TE phonation and fluoroscopic dimensional parameters.

### Manometry

Manometry studies of PE biomechanics in laryngectomees have been performed to investigate the pressure features of the PE segment that may influence TE phonation. Takeshita *et al*.^16^ found the intraluminal pressure (mmHg) at rest and during TE phonation to be 13.1 and 25.5 in good, 17.55 and 36.41 in moderate, and 4.44 and 40.46 in poor TE speakers. This study suggested that the difference in pressure between rest and phonation should be small for efficient TE phonation. Aguiar-Ricz *et al*.^23^ used manometry to compare the pressure of the upper oesophageal sphincter (UOS) at rest and phonation between successful and unsuccessful TE speakers. They found that at rest, the mean UES pressure was 11.83 mm Hg for successful esophageal speakers and 9.92 mm Hg for unsuccessful esophageal speakers and with no significant difference between groups during phonation. These findings imply that the role of intraluminal pressure of the PE segment has not yet been fully understood. The key factors including the pressure gradient across the vibrating segment (ΔP) and PE geometry and their associations with TE phonation have not yet been systematically investigated. This can be examined through use of high-resolution pharyngeal manometry (HRM) combined with concurrent videofluoroscopy, which provides fast signal acquisition and accurate biomechanical measurements during TE phonation.

### Acoustic voice analysis

Acoustic analysis of a recorded sound signal is often used because of the ease and non-invasive nature of sound recording in clinics and correlation with perceptual voice measures^24^. Objective analysis of sound signals including fundamental frequency, frequency and pitch perturbation, spectral characteristics and relative intensity analysis can be obtained using tools such as the Computerised Speech Lab (CSL)^25^ and Praat^26^.

To ensure accurate and meaningful acoustic analysis, signal typing must be undertaken to identify the level of noise in the signals. In 1995 Titze^27^ classified acoustic signals into three types. Type 1 signals were nearly-periodic that did not show qualitative changes and strong modulations or subharmonics (i.e. the energy level of these components, if present, were below that of the fundamental frequency, f0). Type 2 signals contained qualitative changes (e.g. bifurcations) or modulations and subharmonics with energy level approaching that of f0. This signal type did not have an obvious single f0 in the signal. Type 3 signals contained no obvious periodic structure. This signal type was further defined by Sprecher *et al*. as chaotic with a finite dimension^28^. Sprecher *et al*.^28^ also added Type 4 signals i.e. signals that contained an infinite dimension; In spectrograms, there was smearing of energy across a wide range of frequencies similarly to broadband white noise.

It has been proven that analysis of perturbation in voice signals dominated by chaotic and stochastic noise is unreliable^28,29^. For example, Type 3 and Type 4 signals^28^ have been identified as being inappropriate for analysis of perturbation (shimmer and jitter) and harmonics-to-noise ratio (HNR). In recent times, a new measure, cepstral peak prominence (CPP) and its smoothed measure (CPPS)^30^ have been used to analyze Type 3 and 4 signals as it is not dependent on f0 tracking^31^. The CPP is measured as the difference in amplitude between the cepstral peak and the value on a linear regression line directly below the peak relating cepstral frequency (i.e. *quefrency*) to cepstral magnitude^31^. The CPP is able to provide a reliable assessment of dysphonia for voice with high levels of noise^32,33^ and has been demonstrated to have a strong correlation with TE voice quality^34^. In laryngeal phonation, it has been shown that CPP values can vary across vocal tasks^35^, intensity^36^, and acoustic analysis programs^37^.

Acoustic features, such as the maximum phonation time, voice sound intensity, fundamental frequency, perceptual evaluations, and HNR have been well studied in this population^8,38–40^. These studies reveal that in spite of a significant difference from normal laryngeal phonation, TE phonation is the closest to normal phonation compared with other alaryngeal rehabilitation methods^41^.

### Measurement of diameter of the PE segment

The recently developed impedance based Endolumenal Functional Lumen Imaging Probe (EndoFLIP) positioned in the PE segment offers a more direct and precise measurement of the PE geometry^42^. EndoFLIP has been used as a novel technology to study the oesophagogastric junction/lower oesophageal sphincter to measure the outcome of fundoplication and Heller’s myotomy surgeries^43^, to determine the consistent stoma size during gastric banding^44^, to examine oesophageal narrowing due to stenosis^45^, and to assess mechanical competence of the gastroesophageal junction^46,47^. No study has used this method to measure the PE diameter.

To date, no studies have systematically investigated the factors that influence the quality of TE phonation using the combination of the self-evaluation, fluoroscopy, pressure evaluation and acoustic analysis. Therefore, the aims of this study were: (1) to describe the features of TE phonation using patient’s self - rating, acoustic analyses, and dynamic manometric measurements; (2) to investigate the relationship between the patient’s self-rating of satisfaction toward their TE phonation and acoustic measurements and dynamic manometric characteristics during TE phonation; and (3) to examine the effects of changes in biomechanical properties of the PE segment on TE acoustic results.

## Materials and methods

### Ethical approval

The study protocol was approved by the Human Research Ethics Committee of the South Eastern Sydney Local Health District of New South Wales Health (HREC/12/POWH/452). Written informed consent was obtained from all participants to participate in this study. The study was implemented in accordance with relevant ethical guidelines and regulations. The measurement procedures used in this study conformed to the standards set by the latest revision of the Declaration of Helsinki.

### Participants

Fifteen patients participated in this study with mean age of 67 (standard deviation, SD = 7; range = 55 to 77), 14 were male, one was female. Patients were recruited through the Departments of Gastroenterology, Speech Pathology and Radiation Oncology at St George Hospital, and the Laryngectomee Association of New South Wales, Australia. Patients were only included if they had undergone total laryngectomy surgery at least 12 months prior to this study and were communicating using TE phonation at the time of the study. Patients were excluded if they had any history of local tumour recurrence or any neurological disorder potentially associated with dysphagia, such as a prior cerebrovascular accident, Parkinson’s disease, or myopathy.

### Study procedures

#### Pressure measurement of vibrating PE segment

High resolution manometry (HRM) combined with concurrent videofluoroscopy^48^ was used to investigate the biomechanical properties of the PE segment during TE phonation. With participants seated upright, the manometry catheter (Unisensor USA Inc., Portsmouth, NH, USA), with diameter 3.6 mm incorporating 25 solid-state pressure sensors at 1-cm spacing, was inserted transnasally to span the UOS after topical anaesthesia (lignocaine 10%). Fluoroscopic videos were acquired (MultiDiagnost Eleva; Philips, Best, The Netherlands) and recorded concurrently with HRM using an MMS Solar GI system (Software Version 8.21o; MMS, Enschede, The Netherlands).

**Figure 1:**
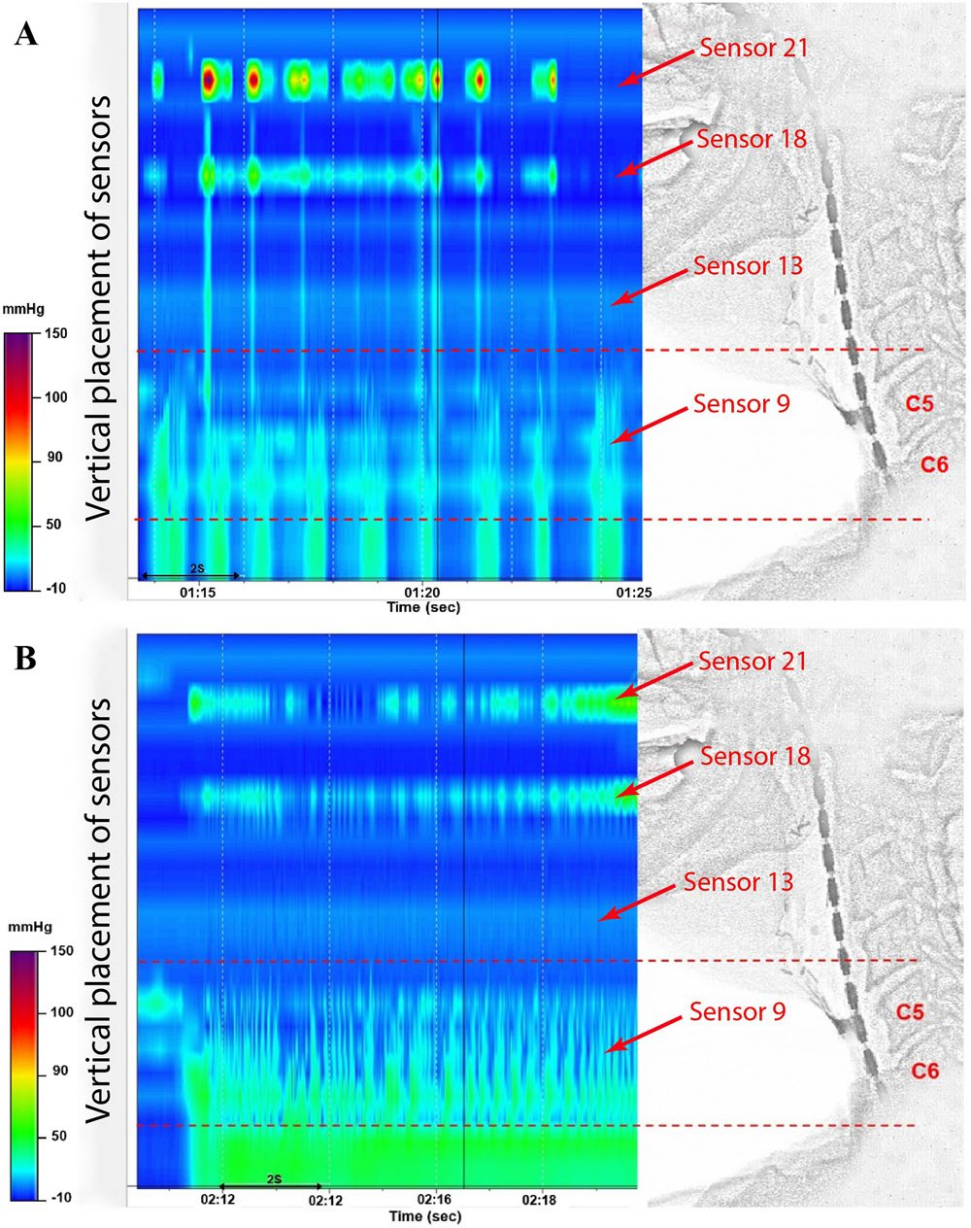
HRM with concurrent videofluoroscopic recording during TE phonation. Patients counted steadily (**A**) and then phonated the prolonged/a/with the stoma closed (**B**). The vertial axis displays the position of the pressure sensors, whilst the colour indicates the air pressure in mm/Hg. From the bottom of the manometric traces and the fluoroscopic images, sensor 9 was located at the mid of hypopharynx and the UOS; sensor 13 at the superior hypopharynx; sensor 18 at the base of tongue and sensor 21 at the soft palate.

Participants swallowed 2 mL of EZ-HD barium (Bracco UK Limited, Woodburn Green, High Wycombe, UK) to enhance visualization of the PE segment before phonation. Participants were then required to take a deep breath and count numbers steadily in one breath and then phonate a prolonged/a/with the stoma closed with videofluoroscopic examination. The anatomical locations of interest were identified by locating the corresponding sensor positions in the fluoroscopic images. The PE pressure gradient (ΔP) was calculated as the difference between the pressure in the upper oesophagus and a point 2 cm above the vibrating PE segment during prolonged/a/(Fig. 1).

Figure 1 shows a representative recording of HRM and concurrent videofluoroscopy during TE phonation. By counting the HRM sensors on the fluoroscopic cine-loops, pressure changes at several anatomical positions of interested were localised during phonation. Pressurisation zones at sensors 21 and 18 respectively indicated the closure of the soft palate and the movement of the base of the tongue during TE phonation. Sensor 13 at the superior hypopharynx measured the atmospheric pressure, which was the reference point to calculate the pressure gradient across the PE segment.

At the bottom of the pressure map, pressurisation of the cervical oesophagus was identified during phonation. Rapid pressure changes demonstrated the anatomical location of maximal vibration of the PE segment, situated between the fourth and sixth cervical vertebrae (C4–C6), and covering the inferior hypopharynx and the UOS (Fig. 1, between the red dashed lines). The movement of the PE wall was also revealed on the concurrent fluoroscopic recording.

#### Diameter measurement of UOS

The Endolumenal Functional Lumen Imaging Probe (EndoFLIP) was used to capture accurate measurement of UOS diameters^19^. The minimal diameter of the EndoFLIP balloon is zero when the lumen is completed obstructed by a passive stricture. Prior to each study, the probe was calibrated at body temperature by filling the bag with 0.2% saline within a calibration block containing a set of cylindrical lumens with their surface areas ranging from 50 to 616 mm^2^ and the pressure transducers were calibrated at 0 and 75 mmHg^47^. The patients, under sedation (fentanyl, midazolam, and propofol), were then kept in the right lateral position to minimise the influence of gravity on the balloon and involuntary contraction of the UOS in response to balloon insertion.

**Figure 2:**
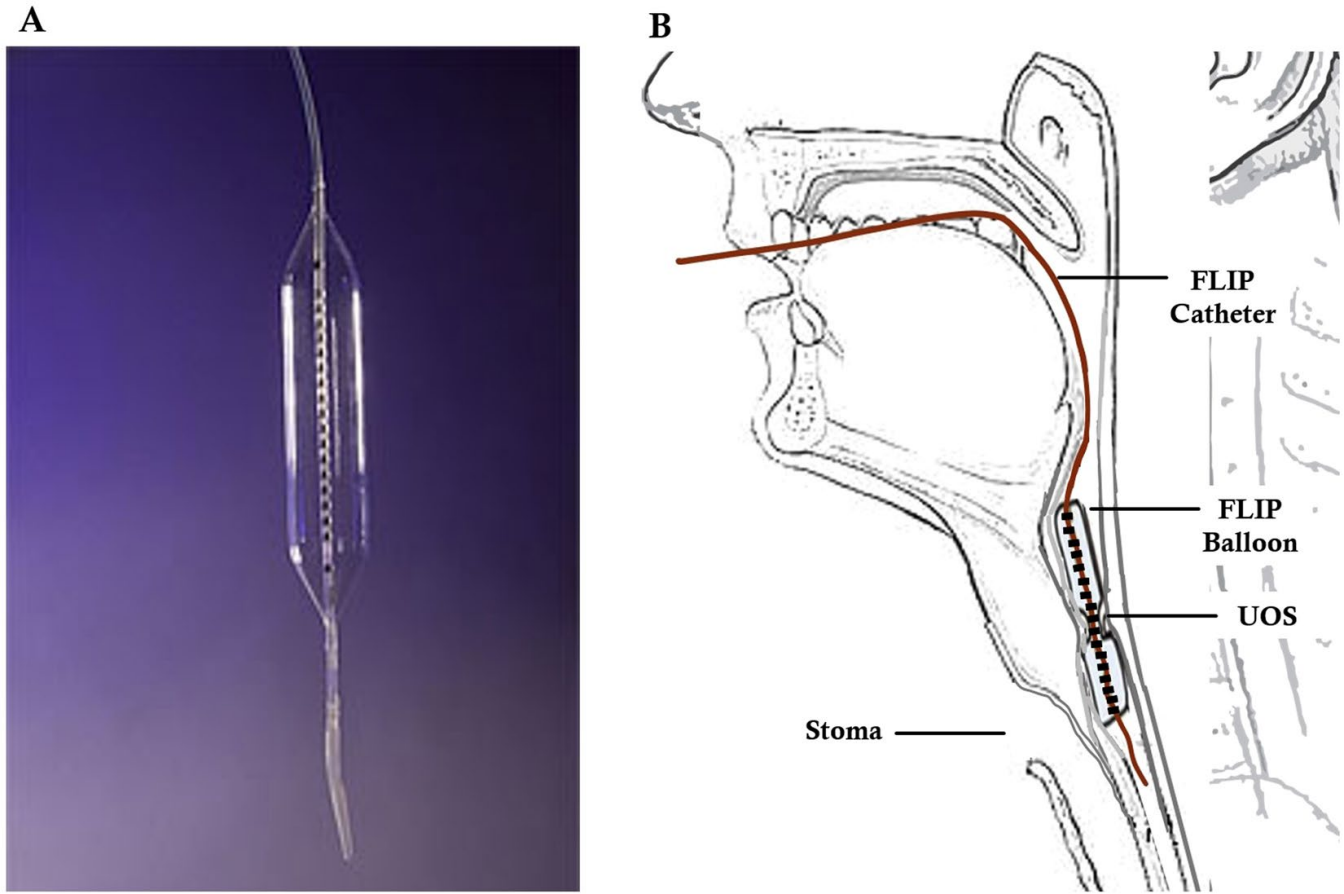
Indications of EndoFLIP position in UOS/PE junction. The EndoFLIP balloon *in vitro* (**A**) was non-compliant and filled with conductive fluid. The position of the balloon *in vivo* (**B**), with an hourglass shape indicating the position of the UOS in the middle of the FLIP balloon.

The EndoFLIP catheter was placed transorally into the oesophagus and withdrawn until the bag was centred at the UOS (Fig. 2B). Bag position was also confirmed by partially filling the balloon (10 mL) and observing an hourglass shape on the EndoFLIP screen. When the catheter was held in place, the balloon was deflated and the patient had a brief (2 min) habituation period. It was followed by 30 mL ramp distension (rate 60 mL/min) to obtain the minimal diameter of the PE segment. The PE segment geometry was monitored in real time to ensure the bag remained in position; a repositioning was necessary if any suspected migrations were observed^47,49^.

To clinically treat UOS strictures and pharyngeal dysphagia, endoscopic dilatation was performed in order to increase the minimal diameter of the UOS. Detailed protocols on the endoscopic dilation can be found in our previous publication^50^. In one patient, the EndoFLIP measurement was repeated after the dilatation.

#### Self-rating of TE voice impairment

The subjective assessment of voice impairment was obtained by using a validated questionnaire known as the Voice Symptom Scale (VoiSS)^51^. The “Impairment” subscale of the questionnaire (VoiSS-I) was selected as the tool for assessing the degree of self-reported satisfaction towards TE phonation. This scale was selected as it had the most rigorous development process compared with other self-rating scales^52^. Previous research has found it to have high sensitivity, specificity, and efficiency in rating the impact of voice problem on the patient’s life^53^. This scale is also simple for patients to use.^51^. The VoiSS was regarded as psychometrically the most robust and extensively validated self-report voice measure^54^.

#### Acoustic analysis of TE voice

The acoustic recordings of TE voice were collected using the CSL Model 4500^25^ at least one week after the UOS diameters measurement to avoid possible effects of this procedure on the PE segment mucosa e.g. oedema that could affect TE phonation. A cardioid (directional) microphone attached to the CSL was placed 20 cm horizontally from the mouth to record the TE voice^55^. Sitting in the upright position, patients were required to take a deep breath, then cover the stoma, and initially count steadily at one number per second. After the initial count step, another deep breath was taken and patients were asked to phonate the vowel/a/as long as possible with the stoma covered. The patient was required to repeat the task three times. The voice signal was recorded at 44.1 kHz sampling rate and saved in *.wav file.

The Praat acoustic analysis program (version 5.1.02)^56^ was used to generate spectrograms of the prolonged/a/using settings described in Sprecher *et al*.^28^ as follows: Hamming window, window length = 50 ms, time step = 0.002 seconds, frequency step = 5 Hz, and a dynamic range of 40 dB. Signal typing was performed visually by the sixth author, by comparing the spectrograms to the exemplar signal types described in Sprecher *et al*.^28^.

CPP (in decibel, dB)^57^ was measured using the *Analysis of Dysphonia in Speech and Voice* (ADSV)^58^ and relative intensity (dB) was measured using the intensity analysis function. CPP was used as it has been recommended in the assessment of voice quality not suitable for more traditional analysis such as noise-to-harmonic ratio, jitter and shimmer^59^. Previous research has found a strong correlation between this measure and TE voice quality^34^. Acoustic analyses for these measures were performed using the whole vowel duration. The mean vowel duration (second, s) was 5.9 s, SD = 3.8, minimum = 2.0 s, maximum = 14.3 s. The final acoustic value for each measure was averaged across the three trials. This study only used this vowel to allow correlation calculation with other dynamic measurements which were also analysed on this vowel.

### Statistical analyses

Statistical analyses were performed using Prism version 4.0 for Windows^60^. Descriptive statistics was used to describe biomechanical and voice measures. Linear regression analysis was used to assess the relationship between ΔP and minimal diameter of the PE segment and TE voice impairment (VoiSS-I), CPP, and relative intensity. The relationship between VoiSS-I scores and acoustic measures (CPP and relative intensity) was also examined using linear regression. In all statistical calculations, a significance level of p < 0.05 was used. Bonferroni correction was not implemented given the preliminary and exploratory nature of this study.

## Results

### Pressure characteristics of PE segment

**Figure 3:**
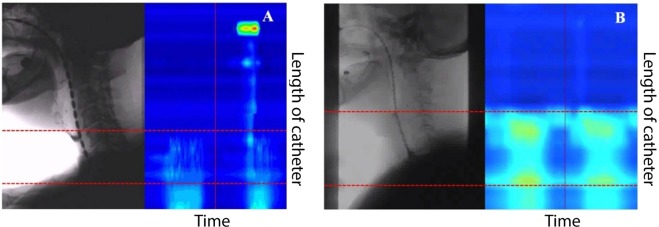
HRM and fluoroscopic features of successful (**A**) and failed (**B**) TE phonation. Dashed lines show the lower and upper boundaries of the PE segment. The colour map represents pressure levels.

Fourteen patients were able to undergo the manometry protocol. By counting the pressure sensors from the fluoroscopic image (Fig. 1), the location of the vibration segment during phonation could be identified over a region covering the inferior hypopharynx and the UOS (sensor 9), corresponding to the cervical vertebrae C4 – C6. Additionally, the soft palate was closed during phonation (sensor 21), with movement of the base of tongue (sensor 18) and unpressurised superior hypopharynx (sensor 13). Figure 3 shows HRM examples of successful (Fig. 3A) and unsuccessful (Fig. 3B) TE phonation with insertion of voice prosthesis along with concurrent videofluoroscopy. Fluoroscopically, in successful TE speakers rapid movement of the PE wall was observed from the fluoroscopic video, whereas in unsuccessful TE phonation no such rapid movement was observed. Manometrically, successful TE speakers demonstrated oesophageal pressurisation and rapid pressure changes at the inferior hypopharynx and the UOS (Fig. 3A, between the red dashed lines). In laryngectomees with failed TE phonation, no rapid pressure changes at the hypopharyngeal region and UOS were present despite oesophageal pressurisation (Fig. 3B). From HRM examination, the ΔP was averaged across 10 data point for each patient. The average ΔP (n = 14) during TE phonation of/a/varied from 15.0–78.0 mmHg across patients (Table 1).

**Table 1:** Descriptive statistics of all measures in the present study.

Parameters	n	Mean (95%CI)	SD	Median	Min	Max	25%-75% percentile
F0 (Hz)	12	213.9 (185.5–42.4)	44.8	222.2	145.1	269.8	170.4–252.2
Intensity (dB)	12	66.4 (60.7–72.1)	9.0	65.45	52.7	83.4	60.3–73.4
CPP (dB)	12	1.9 (0.9–2.8)	1.4	2.01	0.3	4.8	0.6–2.6
VoiSS-I	15	31.6 (24.4–38.8)	12.9	31	9	48	20–44
ΔP (mmHg)	14	44.5 (33.8–55.1)	18.5	41.5	15.0	78.0	33.8–55.1
UOS diameter (mm)	7	10.2 (8.0–12.4)	2.4	10.1	6.5	13.7	8.4–12.1

CI, confidence interval; SD, standard deviation; CPP, cepstral peak prominence; UOS, upper oesophageal sphincter; dB, decibel; Hz, hertz.

### UOS diameters

**Figure 4:**
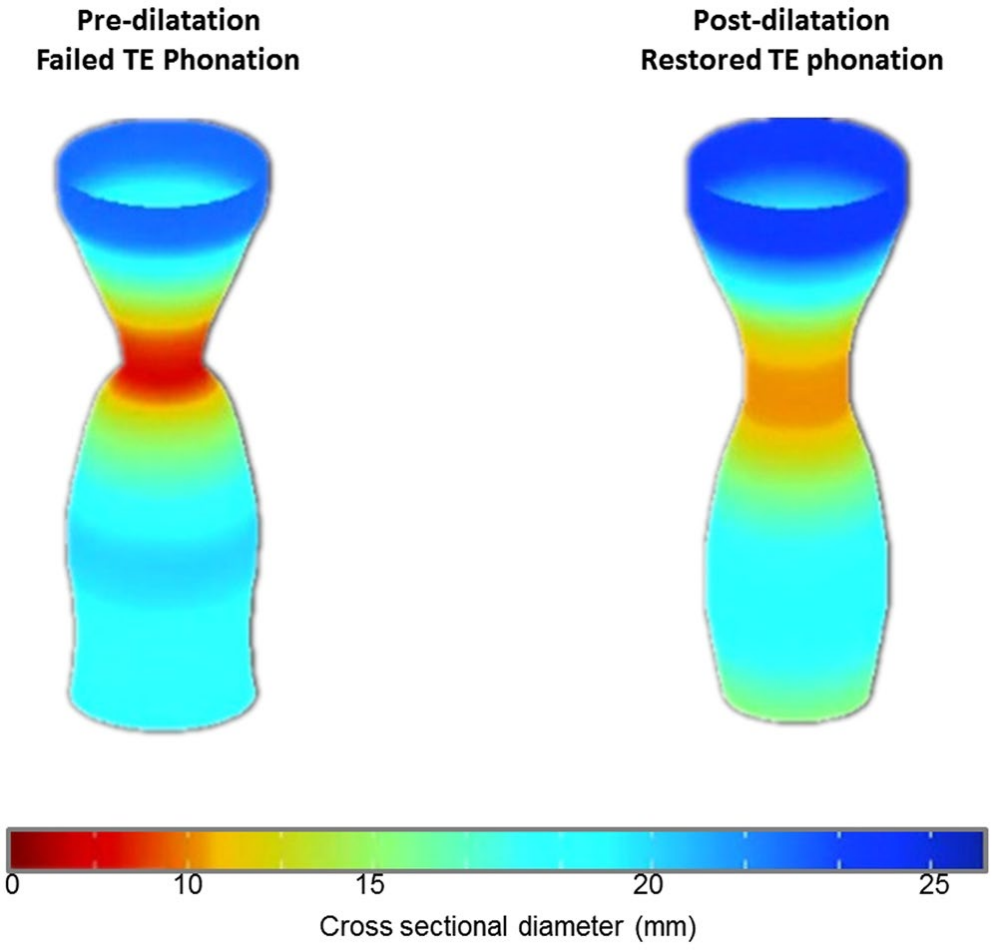
Minimal diameter of UOS and TE phonation.

Table 1 shows the mean, SD and other statistical parameters of the minimal diameter of the PE segment obtained from seven laryngectomees who underwent voice recordings. The UOS minimal diameter was obtained while the patient did not phonate. Two laryngectomees in the study initially with no TE phonation, even with the insertion of a voice prosthesis, underwent endoscopic dilatation for treating pharyngeal dysphagia. After dilatation, the patients were able to communicate using TE phonation. In order to understand this phenomenon, endoscopic dilatation was performed on another laryngectomee with failed TE phonation despite the insertion of a voice prosthesis. Minimal diameters of the PE segment (minimal UOS diameters) pre- and post-dilatation were measured by EndoFLIP. Post-dilatation, the minimal UOS diameter increased from 6.4 mm to 7.3 mm (Fig. 4) and the patient was capable of TE phonation.

### VoiSS and acoustic findings

VoiSS questionnaires were completed by 15 participants with the mean (SD) score of the VoiSS-I subscale being 31.6 (12.9).

In 12 patients who had voice recordings, 2 had Type 2 signals, 6 had Type 3 signals, and 4 had Type 4 signals (see Appendix). Signal type 4 is dominated by stochastic noise with smearing of energy across a wide range of frequencies^28^. Given these signal types in the voice recordings, f0 was not measured. The vowel CPP of the TE voice ranged from 0.3 dB to 4.8 dB, with a mean (SD) value of 1.9 (1.4) dB.

### Relationship between biomechanics and voice measures

**Figure 5:**
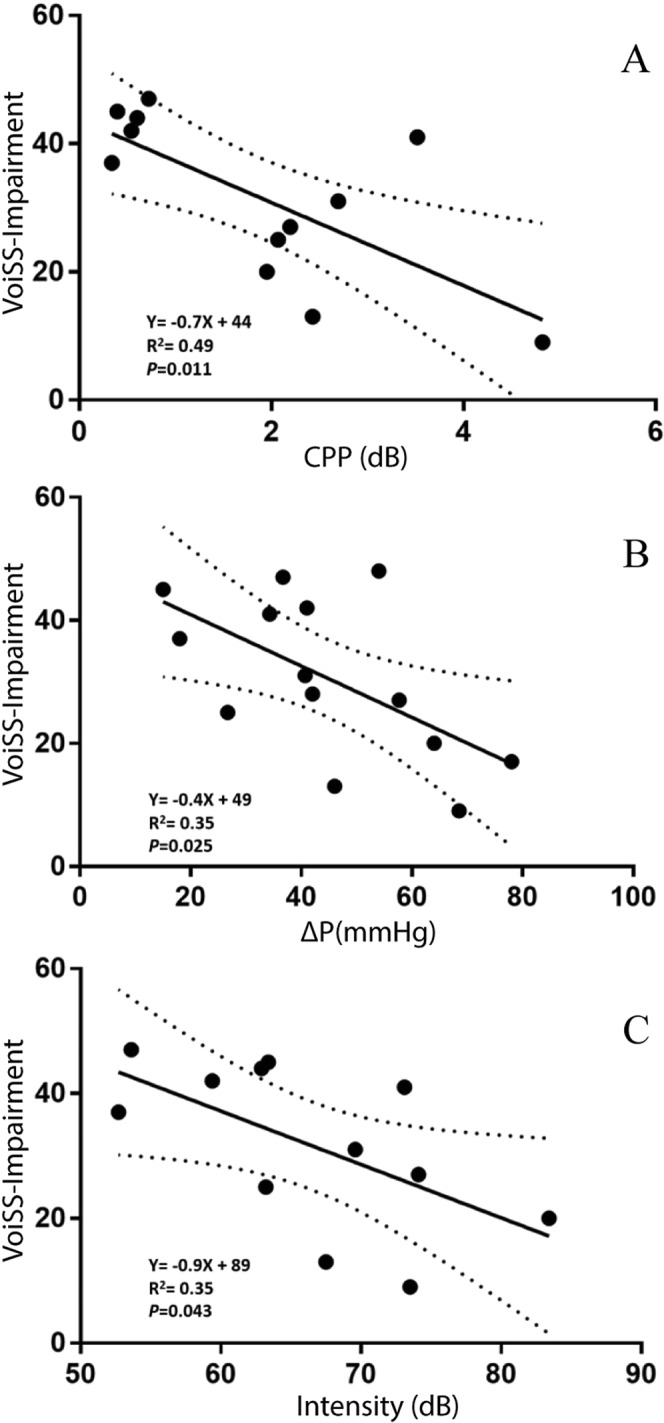
High CPP, ΔP and intensity of the voice indicating satisfactory TE phonation.

Linear regression was used to determine whether biomechanical measures were significant predictors of voice outcomes in TE phonation. Figure 5 shows linear regression lines representing the relationship between CPP, ΔP, and relative intensity and VoiSS-I scores. This figure showed that VoiSS-I scores were statistically significantly predicted by CPP (Y = −0.7X + 44; R^2^ = 0.49; p = 0.011), ΔP (Y = −0.4X + 49; R^2^ = 0.35; p = 0.025), and relative intensity (Y = −0.9X + 89; R^2^ = 0.35; p = 0.043). These regression equations showed that satisfactory TE phonation (i.e. low VoiSS-I score) was associated with a high CPP (Fig. 5A), high ΔP (Fig. 5B), and high intensity (Fig. 5C).

**Figure 6:**
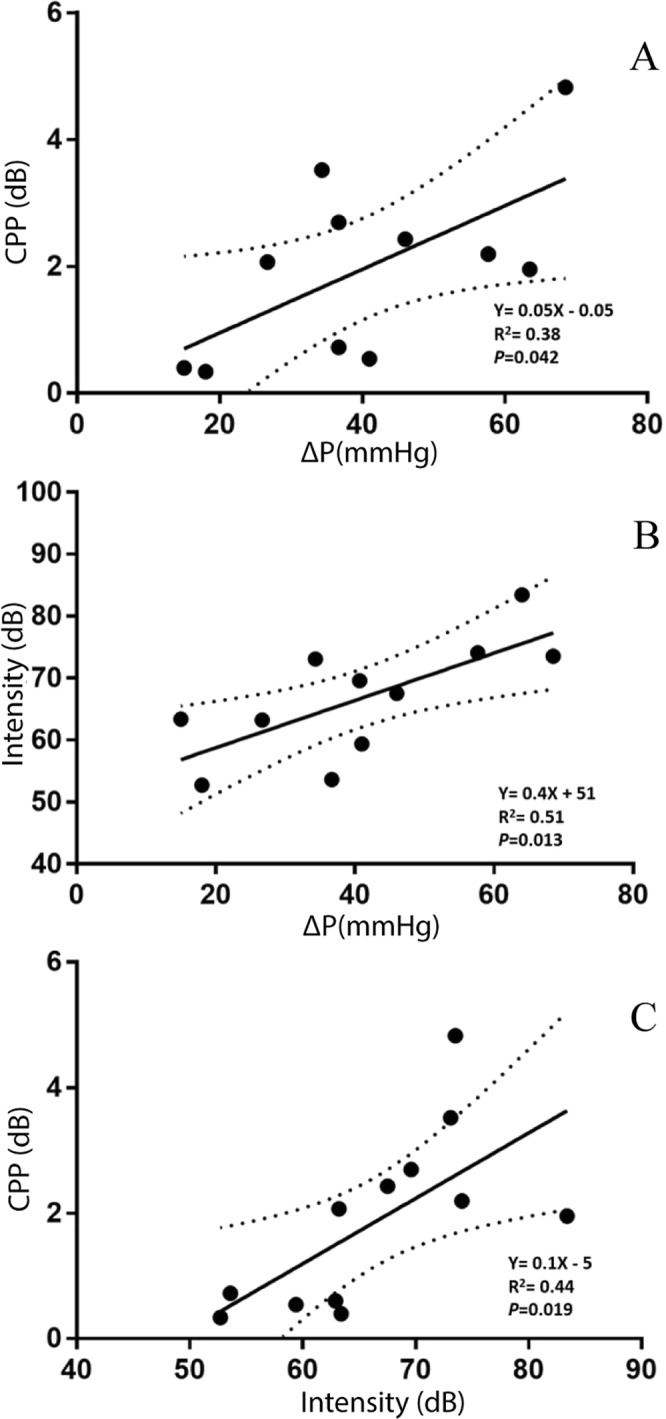
Relationship between ΔP, intensity and CPP during TE phonation.

Data from 11 laryngectomees who had both HRM and TE voice recording were used to calculate the linear regression to evaluate the relationship between ΔP and acoustic measures. The results showed that ΔP was a significant predictor of both CPP (Fig. 6A: Y = 0.05X −0.05; R^2^ = 0.38; p = 0.042) and relative intensity (Fig. 6B: Y = 0.4X + 51; R^2^ = 0.51; p = 0.013) of TE phonation. Larger ΔP was associated with higher CPP and greater intensity. In addition, relative intensity was also a significant predictor of CPP (Fig. 6C: Y = 0.1X − 5; R^2^ = 0.44; p = 0.019), that is, higher intensity would result in higher CPP in TE phonation.

**Figure 7:**
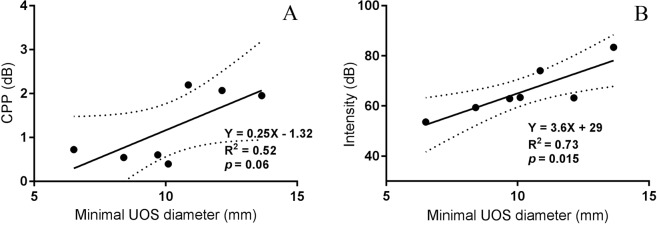
Relationship between minimal diameter of the UOS and CPP and intensity of TE phonation.

Figure 7 shows regression lines and equations representing the relationship between the minimal UOS diameter and CPP and relative intensity of TE phonation. Although a large minimal UOS diameter tended to be associated with a high CPP result, no significant linear relationship between these two measures was found (p = 0.06, Fig. 7A). The minimal UOS diameter was a significant predictor of relative intensity (Y = 3.6X + 29; R^2^ = 0.73, p = 0.015); the larger the UOS diameter, the greater intensity of TE phonation (Fig. 7B).

## Discussion

Understanding the characteristics of the PE segment that determine successful TE phonation is important in the rehabilitation of phonation in laryngectomy patients. This study was an attempt to correlate patient’s satisfaction towards TE phonation and acoustic characteristics of TE phonation to a number of biomechanical measures of the PE segment collected in the context of clinical examination and treatment of PE stricture. The methods used in this study include HRM, videofluoroscopy and EndoFLIP, which provided data on location of the PE segment, intraluminal pressure inside the PE segment, and minimal UOS diameter. The combined assessment was deemed necessary to provide a comprehensive assessment of the PE segment biomechanics.

### Pressure characteristics of the vibrating PE segment

The simultaneous use of videofluoroscopy and manometry^61^ is useful in providing information on the location, pressure, and function of the PE segment. To our knowledge this is the first study to combine HRM and videofluoroscopy to identify the vibrating PE segment during phonation in laryngectomees. The segment was observed to correspond to C4–C6, which is the location of the UOS and inferior hypopharynx. This was higher than the previously reported C5 to C7^9^. It is possible that a larger number of sensors would allow detecting the parts of PE segment that can be otherwise missed if a smaller number of sensors are used. This is an advantage of HRM in examining PE function.

Two major phenomena were observed. Firstly, HRM and videofluoroscopy showed pressurisation and rapid pressure changes in this segment, which were not observed in patients with unsuccessful TE phonation. This showed the essential nature of the vibration of the PE walls on TE voice production. This further supported a previous view that TE phonation should be regarded as an aerodynamic-myoelastic rather than merely aerodynamic event^62^. However, voluntary control over this may not be similar to that in laryngeal phonation.

Secondly, the present study showed that sufficient ΔP is essential in generating satisfactory TE voice in total laryngectomees (i.e., lower voice impairment self-rating and improved acoustic outcomes). There was a wide range of pressure gradients across the PE segment during intelligible phonation (between 15.0 and 78.0 mmHg across patients), implying a wide tolerance in tonic pressure following laryngectomy. To our knowledge, this is the first study to measure ΔP in laryngectomy patients with TE phonation. In individuals with a normal larynx, an oscillation threshold pressure from the lungs will be required to generate the inferior-superior pressure gradient^2^ across the vocal folds for phonation. This pressure gradient induces fast airflow through the vocal folds, creating pressure changes on the surface of vocal fold tissues, which leads to a self-sustained tissue vibration and sound generation^63^. From both the theoretical basis and our experimental data, the muscular pressure gradient across the PE vibration segment during phonation may be an important factor that determines TE phonation. Measurement of air pressure and flow in the PE segment during TE phonation would be required to investigate this hypothesis. Regression analyses confirmed the relationship between self-rated VoiSS scores and CPP, relative intensity, and pressure gradient across the vibrating segment. Thus, ΔP might be a valuable predictor of satisfactory TE voice production.

The results indicated that the ΔP might be regarded as an indirect estimate of the PE segment resistance and hence TE phonation efficiency. In normal phonation, laryngeal airway resistance is determined by air pressure and airflow rate^64^ both of which can be intentionally controlled for. In TE phonation, assuming that the same rule applies, the resistance within the PE segment would be determined by trans-oesophageal air pressure and airflow rate. Grolman *et al*.^65^ found that, although airflow rate at comfortable TE phonation (167 ml/s) was not significantly different from normal laryngeal phonation, the resistance of the PE segment was significantly higher (198 cm H_2_O/l/s). Given the differences in biomechanical characteristics between the larynx and the PE segment, it is possible that achieving an adequate pressure is the most important strategy for TE speakers to produce an acoustic signal. In laryngeal phonation, the relationship between pressure-flow and the interaction between pressure and flow and vocal fold tissue determines vibratory characteristics^66^ in which the phonation threshold pressure is dependent upon the vocal fold geometry and viscoelastic properties^2^. However, in TE phonation, the PE segment as a vibrator may function differently, making the interaction between pressure-flow and PE tissue more complex. The viscoelastic characteristics of the PE segment can change over time as a result of stricture and scarring, influencing the vibration of the PE segment, associating with a degradation of sound quality or a lack of vibration. These can impede vibration or, in the worst scenario, stop the vibration completely. Reducing resistance of this segment may help return the vibration, for example in cases where botulinum injection or PE dilatation are used to restore TE voicing^67^.

### Diameters of the PE segment

We found that minimal UOS diameters, as measured by EndoFLIP, were a significant predictor of TE voice. Given the applicability of impedance planimetry to measure cross-sectional areas within the oesophagus^45^, using this technique in biomechanically assessing the PE segment is reasonable. Lohscheller *et al*. estimated UOS cross-sectional area from HS endoscopy^68^. In comparison to UOS cross-sectional area estimated by HS endoscopy, EndoFLIP can provide a direct, objective and more accurate assessment of the minimal UOS diameter^69^.

The minimal UOS diameter is an important factor for a successful TE phonation because it may determine aerodynamic resistance. In laryngectomy patients phonating with a TE voice prosthesis, the endo-tracheal phonation pressure and aerodynamic resistance are considerably higher than that in normal laryngeal phonation^65^. There is also substantial power loss at the level of the TE voice prosthesis^65^. The constricted PE segment would further increase the resistance. This study showed that endoscopic dilatation increasing the minimal UOS diameter improved TE phonation in a small sample size. More patient data gathered during a clinical trial is necessary to confirm our observations. A small UOS diameter is potentially correctable by endoscopic dilatation, which is a simple and safe therapeutic option, primarily for treating pharyngeal dysphagia with UOS strictures^70^ but not well-recognised as a tool to improve TE phonation. It should also be noted that for patients with adjuvant chemotherapy and UOS narrowing, multiple dilatation sessions (range: 1–12 sessions; mean: 3 sessions) may be required to achieve a response^71^.

Large minimal UOS diameter was correlated with high intensity, but not better quality of the TE voice in this study. This implies that diameter of the UOS may not be the only factor to determine the quality of TE voice. The two patients with failed rehabilitation of TE phonation underwent endoscopic dilatation to increase the minimal UOS diameters and to treat dysphagia. The capability of TE phonation was subsequently restored. In one patient with failed TE phonation, EndoFlip measured UOS minimal diameters pre- and post- dilation confirmed that the enlarged minimal diameter was associated with the capacity of using TE phonation.

### Voice quality in TE phonation

The voice recordings of laryngectomees in this study had aperiodic signals in which Type 3 and Type 4 signals predominated. In this study, the mean CPP of the TE phonation was 1.9 dB with a range from 0.3 to 4.8 dB. The CPP value in the present study was significantly lower than that in vocally healthy speakers in a study by Madill *et al*.^37^ in which CPP of the same vowel (/a/) obtained by SpeechTool is 17.7 dB (SD = 2.19). The CPP values in this study were also considerably lower than mean CPP values of the/a/vowel from participants at the highest age range (50 years old) measured using ADSV (12.145 dB, SD = 2.044 for males and 10.894 dB, SD = 1.751 for females)^72^. In the literature, only one study has measured CPP from TE phonation^34^ in which the mean CPP measured by SpeechTool from sustained vowel combined with connected speech was 11.45 dB (range = 8.66–15.54 dB). It should be noted that there are factors responsible for the differences in CPP between this study and the literature. It has been found that different software packages produce different CPP results^37,73^. This is due to the different processing algorithms used in each analysis program. Even in the same program and algorithm, different CPP values may result using different settings. Phadke^73^ has pointed out that the output can be different if different versions of the same program (e.g. Praat) stipulate different “default” time and quefrency averaging windows. Additionally, there may be unexplained between-version differences in CPP even when the same settings were used, for example in Praat^73^. Given these facts, future studies on CPP should clearly specify the settings used to extract the data and cross-studies comparisons would be irrelevant if there are differences as discussed above. The low CPP values as observed reflected the degraded sound quality provided by TE phonation as a result of differences in phonation mechanisms compared with normal laryngeal phonation. The physiologic characteristics of the PE segment may not allow voluntary control over TE phonation. Omori *et al*.^6^ found two separate bulges in the PE segment in which the upper bulge, which was made of the thyropharyngeal muscle, was found to be the sound source. They maintained that TE phonation stemmed from thyropharyngeal muscle contraction in harmonization with mucosal vibration in the PE segment. However, unlike laryngeal phonation, these muscular structures of the PE segment are not naturally designed for phonation^74^. Consequently, the modulation of the vibrating tissue with the air flow and pressure may not be as effective as that in laryngeal phonation, creating considerable aperiodicity and noise in the signal as presented by very low CPP as mentioned above.

In this study, we also found that CPP was significantly predicted by intensity (Fig. 6C), which agreed with the literature on laryngeal phonation^36^. Although CPP is considered a robust acoustic measure of voice quality^59^, the findings in this study could not determine whether an increase in CPP resulted from intensity or voice quality of TE phonation. Auditory-perceptual judgements and nonlinear analysis^75^ may be useful in evaluating voice quality independently from intensity.

The negative relationship between CPP and VoiSS scores confirmed the impact of poor voice quality in patients’ satisfaction on TE voice. Our findings are consistent with Robertson *et al*.^76^ in which the mean VoiSS score (Impairment) for laryngectomy patients was 29.8 (SD = 12.5).

### Limitations and future directions

This study had a number of limitations. In acoustic analysis, only a sustained vowel was used. This allowed correlation calculation between acoustic and biomechanical measures and minimized the within-speakers task-specific variations in the acoustic output. However, this may not reflect the actual varying phenomena occurring in connected speech and preclude any generalization to normal speech production. Future studies should use speech tasks for both acoustic and biomechanical measurements.

This study could not assess the degree of aerodynamic power loss and the myoelastic property of the PE tissue. In fact, there may be considerable between-subject variability in these factors, which may affect vocal efficiency, intensity, and voice quality^65^. It is difficult to control for these factors given differences across patients in the power loss due to the prosthesis, the extent of prior surgery and use of different surgical techniques. Therefore, these remain the major challenging factors when studying PE segment and TE phonation. Additionally, this study did not measure aerodynamic parameters e.g. airflow and phonatory pressure. Collecting these would allow comparing biomechanical factors with vocal efficiency of TE phonation, providing more insight regarding what is needed for optimal function of the PE segment in TE phonation.

The sample size in this study was limited and not all patients were included in all analyses. This was due to a range of reasons including patient attrition during the study, difficulties tolerating the procedure and technical difficulties at the time of data collection. This may affect statistical significance level in some of the correlation calculations. A study replicating our protocols using a calculated sample size may clarify the potential correlations in this study e.g. the relationship between UOS diameter and CPP.

Lastly, perceptual analysis of TE phonation was not used in this study. This limited the explanation of acoustic findings. Future studies may combine perceptual and acoustic analyses and biomechanical measurements to provide a better understanding of TE phonation.

## Conclusion

This study found significant correlation between pressure gradient and patient’s voice-related satisfaction and a robust acoustic voice measure, CPP. This suggests that sufficient pressure difference across the PE segment and lower pharynx is needed for efficient TE phonation.

A large minimal diameter tended to result in a higher CPP result, but no significance in this trend was found. Large minimal diameters of the PE segment were associated with higher relative intensity production. As such, a large UOS diameter is a predictor for successful TE voice. In unsuccessful TE phonation patients, endoscopic dilatation increasing the UOS minimal diameter may provide a new approach to treat unsuccessful TE phonation.

## Supplementary information

Spectrograms of all patients in this study
