# Supplementary material for: The relationship between biomechanics of pharyngoesophageal segment and tracheoesophageal phonation
Source: Sci Rep. 2019 Jul 5;9:9722. doi: 10.1038/s41598-019-46223-7 (PMC6611845; doi:10.1038/s41598-019-46223-7)
Supplement: Supplementary file 1 — Spectrograms of all patients in this study [file 41598_2019_46223_MOESM1_ESM.docx]

**The relationship between biomechanics of pharyngoesophageal segment and tracheoesophageal phonation**

**Teng Zhang^1^, Ian Cook^2^, Michał Szczęśniak^2^, Julia Maclean^3^, Peter Wu^2^,**

**Duong Duy Nguyen^4^, Catherine Madill^4^***

St George & Sutherland Clinical School**^1^**, Faculty of Medicine, The University of New South Wales; Department of Gastroenterology**^2^** and Cancer Care Centre**^3^**, St George Hospital; Voice Research Laboratory**^4^**, The University of Sydney; Sydney, Australia

**Appendix**

**Spectrograms of all patients in this study**

**
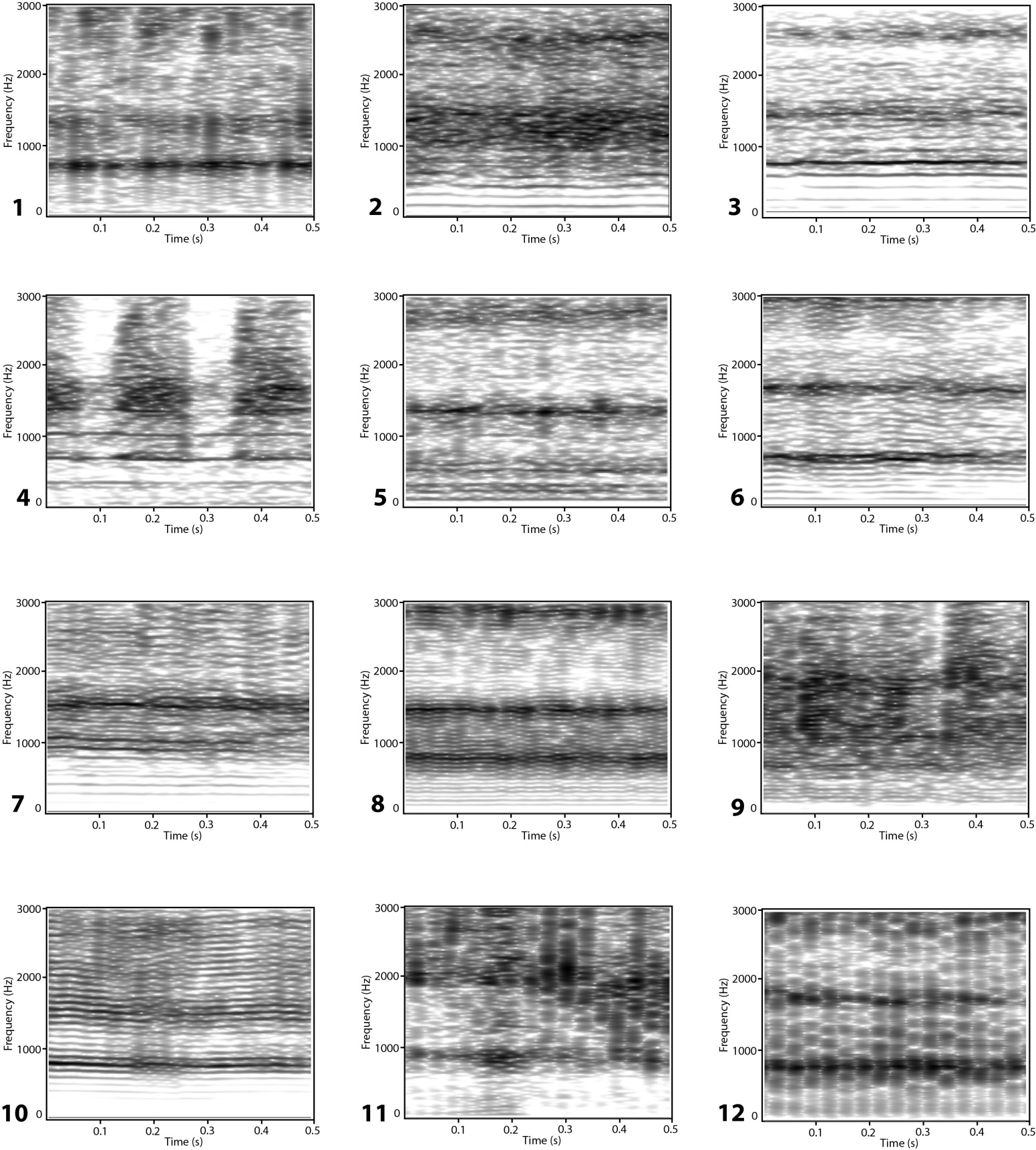
**
